# Sleep Promotion by 3-Hydroxy-4-Iminobutyric Acid in Walnut *Diaphragma juglandis Fructus*

**DOI:** 10.34133/research.0216

**Published:** 2023-09-19

**Authors:** Jian Ji, Yongli Ye, Lina Sheng, Jiadi Sun, Qianqian Hong, Chang Liu, Jun Ding, Shuxiang Geng, Deping Xu, Yinzhi Zhang, Xiulan Sun

**Affiliations:** ^1^ State Key Laboratory of Food Science and Technology, School of Food Science and Technology, National Engineering Research Center for Functional Food, Synergetic Innovation Center of Food Safety and Quality Control, Jiangnan University, Lihu Avenue 1800, Wuxi, Jiangsu 214100, P.R. China.; ^2^ College of Food Science and Pharmacy, Xinjiang Agricultural University, No. 311 Nongda Dong Road, Ürümqi, Xinjiang, Uygur Autonomous Region 830052, P.R. China.; ^3^ Department of Chemistry, Wuhan University, Wuhan, Hubei 430072, P.R. China.; ^4^ Yunnan Academy of Forestry and Grassland, Kunming, Yunnan 650201, P.R. China.

## Abstract

Insufficient sleep can produce a multitude of deleterious repercussions on various domains of human well-being. Concomitantly, the walnut (*Juglans mandshurica*) confers numerous salutary biological activities pertaining to sleep. Nevertheless, the sedative and hypnotic capacities of walnut’s functional constituents remain obscure. In this investigation, we analyzed the sedative and hypnotic components of the walnut *Diaphragma juglandis fructus* and innovatively discovered a compound, defined as 3-hydroxy-4-iminobutyric acid (HIBA), which disrupts motor activity and enhances sleep duration by regulating the neurotransmitters (GABA, DA, etc.) within the brain and serum of mice. Subsequently, a metabolomics approach of the serum, basal ganglia, hypothalamus, and hippocampus as well as the gut microbiota was undertaken to unravel the underlying molecular mechanisms of sleep promotion. Our data reveal that HIBA can regulate the metabolism of basal ganglia (sphingolipids, acylcarnitines, etc.), possibly in relation to HIBA’s influence on the gut microbiome (*Muribaculum*, *Bacteroides*, *Lactobacillus*, etc.). Therefore, we introduce a novel natural product, HIBA, and explicate the modulation of sleep promotion in mice based on the microbiota–gut–brain axis. This study contributes fresh insights toward natural product-based sleep research.

## Introduction

Walnut (*Juglans mandshurica*) is among the most ubiquitous nuts across the globe. It is endowed with a copious amount of unsaturated fatty acids, proteins, polyphenols, and minerals that exhibit vital biological activities such as antioxidation, anti-tumorigenesis, anti-inflammation, cholesterol decrement, blood pressure mitigation, and abatement of the risk for cardiovascular diseases. Previous work has demonstrated that walnut contains sedative and hypnotic components, improving sleep quality [1]. The extract of crushed unripe walnut hulls (*Juglans nigra*) contained depressant agents including 5-hydroxyl-1,4-naphthoquinone (*juglone*), which depressed the movements of leopard frogs, perch, catfish, goldfish, mice, rats, and rabbits [2]. Ellagic acid has also been isolated from walnut hulls (*J. nigra*), and its injection produced a marked sedative effect and augmented the duration of sodium pentobarbital-induced sleep in murine subjects [3]. Walnut consumption increases blood levels of the neurotransmitter melatonin, which in turn affects sleep quality [4]. According to traditional Chinese medicine, *Diaphragma juglandis fructus* (DJF) acts as a sleeping aid by reducing anxiety [5], and DJF extract may shorten sleep latency and prolong sleep duration in mice [6].

As reported, the quality of sleep is regulated by the chemical metabolism in the brain, especially basal ganglia (BG) [7]. BG plays a important role in regulating the sleep–wake cycle via their contribution to a thalamo–cortical–basal ganglia network that oscillates in slow-wave sleep and remains active during rapid eye movement (REM) sleep [8]. Cell body-specific lesions in the BG substructures, the striatum and globus pallidus, increased wakefulness of rats by 14.95% and 45.52%, respectively [9]. The nucleus accumbens of the ventral striatum regulates wakeful behavior through adenosine and dopamine receptors. Control of active phase sleep by striatal adenosine A2A receptors is mediated by parvalbumin neurons in the external globus pallidus (GP) [10]. However, the metabolic mechanism of BG in sleep regulation remains largely unexplored [11].

The brain also works with the microbiota and gut to form an intricate communication network to regulate sleep. The gut microbiome interacts with the hypothalamic–pituitary–adrenal axis to shape the architecture of sheep [12]. The total diversity of gut microbiome, particularly phyla richness of *Bacteroidetes* and *Firmicutes*, was reported to be positively correlated with increased sleep efficiency and total sleep time, while taxa such as *Lachnospiraceae*, *Corynebacterium*, and *Blautia* were negatively correlated with sleep measures [13]. Sleep fragmentation reduced the abundance of *Bacteroidetes*, *Actinobacteria*, and *Bifidobacteriaceae* in humans [14]. Chronic sleep disruption increased the growth of the *Lachnospiraceae* and *Ruminococcaceae* families, and decreased the *Lactobacillaceae* family in mice [15].

In this work, a compound called 3-hydroxy-4-iminobutyric acid (HIBA) is isolated from the DJF, a dry woody membrane inside the walnut. In the mouse model, sedative and hypnotic effects are observed. Using high-resolution mass spectrometry (MS)-based metabolomics of serum, BG, hypothalamus, hippocampus, and gut microbiota analysis, the molecular mechanism of HIBA to promote sleep quality was investigated. HIBA could affect the gut flora (*Flavonifractor*, *Parabacteroides*, *Bacteroides*, *Lactobacillus*) and further regulate the sleep quality of mice by altering the BG metabolism (neurotransmitters, acylcarnitines, purine nucleosides, etc.) via the microbiota–gut–brain axis.

## Results

### Isolation and structure identification of sedative–hypnotic molecule

**Fig. 1.**
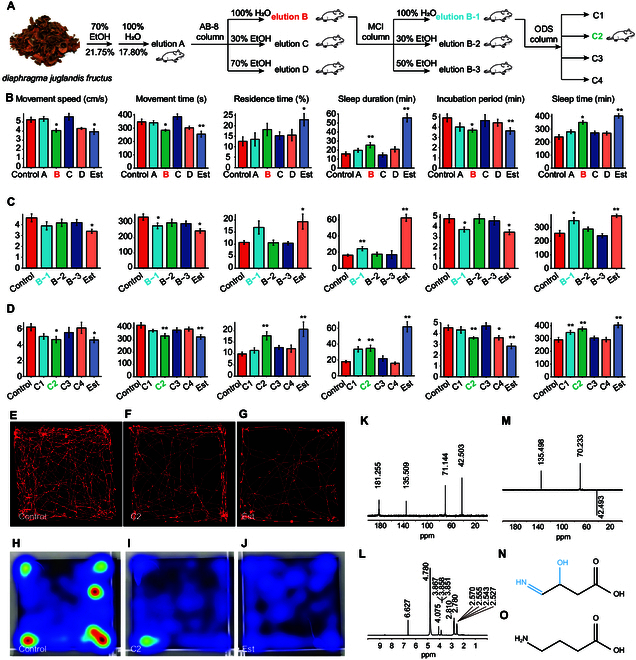
Isolation and structure identification of sedative–hypnotic molecule from DJF through column separation technology. (A) Isolation process of sedative–hypnotic molecule from DJF, and the mouse experiments (*n* = 10) for functional validation of the components, water extraction and alcohol precipitation, separation using MCI column, and separation using ODS column. (B) Barplot depicting the 4 collection fractions (A, B, C, and D) obtained through water extraction and alcohol precipitation. (C) Barplot illustrating the elution fractions (B-1, B-2, and B-3) obtained from fraction B using the MCI column. (D) Barplot showcasing the separated monomer compounds (C1, C2, C3, and C4) obtained from fraction B-1 using the ODS column. Movement speed, movement time, and residence time are 3 factors used to evaluate the sedation status of mice. Sleep duration, latency period, and sleep time are 3 factors used to evaluate the sleep patterns of mice. *P* values were calculated using Student’s two-tailed *t* test. * Indicates a significant difference from the blank group (*P* < 0.05, parametric test); ** indicates that the difference with the blank group is extremely significant (*P* < 0.01, parametric test). Mouse movement track monitoring: (E) control, (F) C2, and (G) Est. Mouse activity heat map: (H) control, (I) C2, and (J) Est. The NMR spectrum and structure of C2: (K) ^13^C NMR, (L) ^1^H NMR, and (M) ^13^C NMR data. (N) Molecular structure of C2. (O) Molecular structure of γ-aminobutyric acid (GABA).

We extracted the active ingredients in HIBA by water extraction and alcohol precipitation, combined with column separation technology, and then gradually verify the extracted active ingredients through mics experiments (Fig. 1A). The water extract of DJF was added to 3 times the volume of anhydrous ethanol, and it was allowed to stand overnight to prepare the mixed extract (elution A). Then, the mixed extract was loaded to an AB-8 macroporous adsorption resin column, and gradient elution was performed with water (elution B), 30% ethanol (elution C), and 70% ethanol (elution D) in sequence. The optimized parameters such as material-to-liquid ratio, column loading, elution time can be obtained in Fig. S1. The sedative–hypnotic activity of the 4 components was studied through animal experiments (Tables S1 and S2). The open field experiment from Fig. 1B shows that, compared with the blank group, both the estazolam (Est) group and elution B significantly reduced the movement speed and movement time of the mice (*P* < 0.05). Among them, the Est group significantly increased the residence time of the mice in the central area (*P* < 0.05). There was no statistical difference in other elution fractions. For the total sleep duration within 12 h after gavage, although each experimental group has an increase in sleep time compared with the blank group but with a *P* value larger than 0.05, only the elution B and Est group showed a significant difference (*P* < 0.01). In addition, the results of sleep experiments induced by sodium pentobarbital showed that the sleep latency was shortened by 24.62% (*P* < 0.05) and 25.88% (*P* < 0.05) in the elution B and Est group, respectively, and the length of sleep was prolonged by 61.49% (*P* < 0.05) and 254.19% (*P* < 0.01). Therefore, elution B is the active part of sedative and hypnotic effects in the distraction tree.

Elution B was further loaded onto a minimal inhibitory concentration (MCI) chromatographic column, and 3 elution fractions using water (elution B-1), 30% ethanol (elution B-2), and 50% ethanol elution (elution B-3) were obtained (Tables S3 and S4). Compared with the blank group, in Fig. 1C, although elution B-1 did not significantly reduce the movement speed of the mice (reduced by 15.87%), it reduced the movement time by 17.17% (*P* < 0.05), and the residence time in the central area increased by 57.85% (*P* < 0.05). The total sleep duration within 12 h after gavage of the elution B-1, B-2, and Est group increased by 36.40% (*P* < 0.05), 11.99%, and 49.77% (*P* < 0.01), respectively, while that of elution B-3 decreased. The elution B-1 and Est group shortened sleep latency by 21.87% (*P* < 0.05) and 27.21% (*P* < 0.05), respectively, and prolonged sleep duration by 49.09% (*P* < 0.05) and 281.30% (*P* < 0.01). The thin-layer chromatography (TLC) diagram of eluted components of MCI column was displayed in Fig. S2A.

To obtain a monomer compound with the sedative and hypnotic activity, elution B-1 with the sleep-aid activity was purified by ODS-A, ODS-AQ, ODS-D, and other chromatographic methods (Tables S5 and S6). Four single-spotted compounds, including C1, C2, C3, and C4, were obtained as shown in the TLC display in Fig. S2B. The mouse model experiment suggested that C2 significantly reduced the movement speed and movement time of the mice (*P* < 0.05). The residence time of the C2 and Est group increased (*P* < 0.01) compared with the blank group. The results of sleep experiments induced by sodium pentobarbital showed that C2 shortened sleep latency by 24.62% (*P* < 0.05) and prolonged sleep duration by 61.49% (*P* < 0.05). From the mouse movement track monitoring (Fig. 1, E to G), we could notice that the density of red lines (movement track) in the C2-treated group is much lower than that in the control group, and the density of red lines in the C2-treated group is like that in the Est group. It indicated that C2 has a good effect on sedation and hypnosis in mice, which is close to the effect of the commercial drug. The mouse activity heat map in Fig. 1 (H to J) also validated the results by visualizing a similar reduction trend of activity in both the C2 group and the Est group.

Nuclear magnetic resonance (NMR) analysis was utilized for structure identification of C2. ^1^H NMR (D_2_O, 500 MHz) spectrum verified a total of 5 hydrogen signals, which are δH6.63 (^1^H, s), 4.08 (^1^H, s), 3.86 (^1^H, s, J = 4 Hz), 2.78 (^1^H, d, J = 15 Hz), 2.55 (^1^H, dd, J = 7.5, 8 Hz), where δH4.07 is the hydrogen signal of oxygen–carbon, and 2.78 and 2.55 are the hydrogen signal of -CH2- (Fig. 1L). The ^13^C NMR spectrum suggested a total of 4 carbon signals, which are δ181.2 (-COOH), 135.5, 71.1, and 42.5 (Fig. 1K). The 135DEPT spectrum shows that δ135.5 is the carbon signal of -CH-. According to the chemical shift, -CH- is connected to the imino group with a double bond, δ70.2 is the oxycarbon signal, and δ42.5 is the carbon signal of -CH2- (Fig. 1M). C2 was annotated as HIBA (Fig. 1N). The structure of HIBA is similar to that of γ-aminobutyric acid (GABA), except that HIBA has one more -OH group compared to GABA. The carbon-4 position of HIBA is connected to an imino group (-NH=), while the carbon-4 position of GABA is connected to an amino group (-NH_2_) (Fig. 1O). It indicates that HIBA may have a similar molecular function to GABA.

### Neurotransmitters in brain and serum

**Fig. 2.**
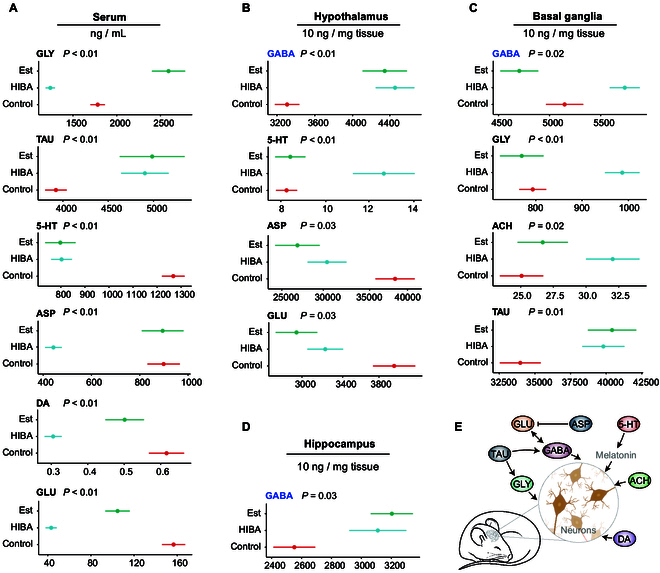
Levels of neurotransmitters in the brain and serum. Levels of neurotransmitters in (A) serum (*n* = 8), (B) hypothalamus (*n* = 8), (C) BG (*n* = 8), and (D) hippocampus (*n* = 8) in the control, HIBA, and Est group. GLY, glycine; TAU, taurine; 5-HT, 5-hydroxytryptamine; ASP, aspartic acid; DA, dopamine; GLU, glutamic acid; ACH, acetylcholine. In serum, the unit of neurotransmitters is ng/ml; in the brain tissue, the unit of neurotransmitters is ng/mg. (E) Relationship between 8 neurotransmitters. *P* values were calculated using Student’s two-tailed *t* test, comparing the HIBA group with the control group.

The ultra-high performance liquid chromatography coupled with hybrid triple quadrupole linear ion trap mass spectrometer (UHPLC-q-TRAP) MS was utilized to determine the contents of 8 neurotransmitters in mouse serum and brain tissue, including monoamines [5-hydroxytryptamine (5-HT), dopamine (DA)] and amino acids [excitatory transmitters such as glutamic acid (GLU) and aspartic acid (ASP); inhibitory transmitters such as GABA, glycine (GLY), and taurine (TAU)] (Data S1). Figure 2 displays the neurotransmitters with *P* < 0.05 between control and HIBA in the brain and serum. In terms of serum (Fig. 2A), HIBA significantly reduced the content of GLY (*P* < 0.01), 5-HT (*P* < 0.01), ASP (*P* < 0.01), DA (*P* < 0.01), and GLU (*P* < 0.01), and significantly increased the content of TAU (*P* <0.01). The trend was basically the same as that of the Est group, except for ASP and GLY. In the brain, neurotransmitter differences were mainly found in hypothalamus (Fig. 2B) and BG (Fig. 2C). In the hypothalamus, HIBA significantly increased the content of 5-HT, GABA, and acetylcholine (ACH) (*P* < 0.05), but significantly reduced the content of GLU and ASP (*P* < 0.05). In BG, GABA (*P* = 0.02), GLY (*P* < 0.01), ACH (*P* = 0.02), and TAU (*P* = 0.01) significantly increased in the HIBA group, compared with the control group. However, in the hippocampus, only GABA (*P* = 0.03) increased significantly in the HIBA group (Fig. 2D). Figure 2E reveals the relationship between the neurotransmitters detected in the experiment and brain neurons; this, to a large extent, governs sedative and hypnotic action. In these 3 brain regions, we can observe that the levels of GABA are elevated, which does not rule out the possibility that HIBA is metabolically converted into GABA in the gut or in other organs.

### HIBA impact on the mouse brain metabolome

**Fig. 3.**
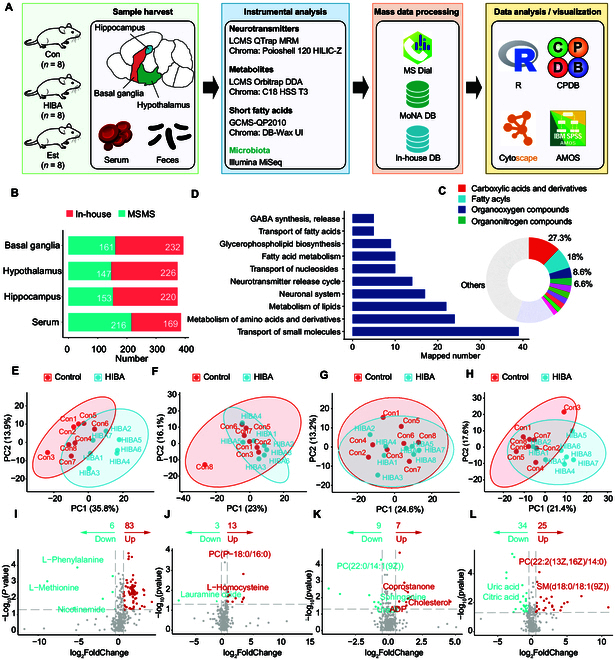
The mouse brain metabolome affected by HIBA. (A) Serum (*n* = 8), brain tissue (*n* = 8), and metabolomics and feces (*n* = 5) microbiota workflow. (B) Number of annotated metabolites by metabolome assay in serum and brain tissues (hippocampus, hypothalamus, and BG). (C) Chemical composition of the mouse brain metabolome using ClassyFire categories to classify the metabolite diversity of all annotated metabolites across assays. (D) Mapping annotated metabolites to pathways. Top 10 mapped pathway-based sets shown from a total of 226 pathways covered by ConsensusPathDB. PCA score plot of (E) serum, (F) hippocampus, (G) hypothalamus, and (H) BG between control and HIBA group; volcano plots of (I) serum, (J) hippocampus, (K) hypothalamus, and (L) BG between the control and HIBA group. The number of up-regulated and down-regulated metabolites was annotated in the figures for statistical analysis, and the statistical criteria used for selection were a *P* value less than 0.05 and an absolute FC greater than 1.5.

To obtain an overview of the HIBA impact on the brain metabolome, UHPLC-HESI-HRMS (high-resolution mass spectrometry) was utilized for untargeted metabolomics on hippocampus (Data S2), hypothalamus (Data S3), BG (Data S4), and serum (Data S5) from 8 control and 8 HIBA-treated C57BL/6 mice. We detected and aligned ion features into master peak tables for each sample type based on MS1 full-scan data. LC-MS/MS raw data were processed by MS-DIAL [16] and annotated by matching to the MS/MS spectra from MassBank of North America (MoNA). Moreover, we used a local database, based on retention times and precursor ions, to identify metabolites. The approximate analysis flow is shown in Fig. 3A. We performed the analysis of the number of metabolites identified in the different samples, as shown in Fig. 3B, generating annotated metabolite numbers ranging from highest 393 for BG to lowest 373 for hippocampus. We defined metabolites into chemical superclasses using the ClassyFire classification system [17] (Fig. 3C), including carboxylic acids and derivatives (27.3%), fatty acyls (18%), organooxygen compounds (8.6%), and organonitrogen compounds (6.6%). We explored the pathway coverage of the annotated brain metabolome by querying annotated metabolites in consensus PathDB [18], comprising 325 pathway-based metabolite sets (Data S6). The top 10 most important pathways are illustrated in Fig. 3D, indicating a sufficient breadth of pathway modules to interpret metabolic changes in brain regions. The principal components analysis (PCA) plot in Fig. 3E to H indicated substantial differences in metabolic signatures between the HIBA and control group. Metabolic profiles of HIBA-treated mice differed in the 3 parts of brain and the blood. The most significant differences were found in BG, followed by differences in serum, and in hippocampus and hypothalamus, almost no difference between HIBA and control groups was seen. We illustrated the metabolites with statistical differences in the form of volcano plots (Fig. 3I to L), and the screening principle was that the *P* value was less than 0.05 and the log_2_FC (fold change) value was greater than 0.58 or less than −0.58. In BG, there were 83 up-regulated metabolites and 6 down-regulated metabolites; in the serum metabolome, there were 25 up-regulated metabolites and 34 down-regulated metabolites; however, in hippocampus and hypothalamus, there were few significant changed metabolites. This result is also consistent with the characteristics shown by PCA. The results guide us to focus on BG in the follow-up in-depth research. HIBA may improve sleep quality by affecting or interfering with the metabolism of BG. In the blood metabolome, we can also clearly observe that HIBA caused some changes in serum metabolites. It can be preliminarily judged that HIBA may affect the sleep quality of mice by changing metabolites in blood and BG metabolism. This conclusion was also verified in the previous neurotransmitter analysis results. The change in the brain and serum neurotransmitter levels may be related to changes in the level of intestinal flora. In the follow-up research, we will focus on whether HIBA interferes with the intestinal flora, then affects brain metabolism through blood metabolism, and promotes changes in sleep quality.

**Fig. 4.**
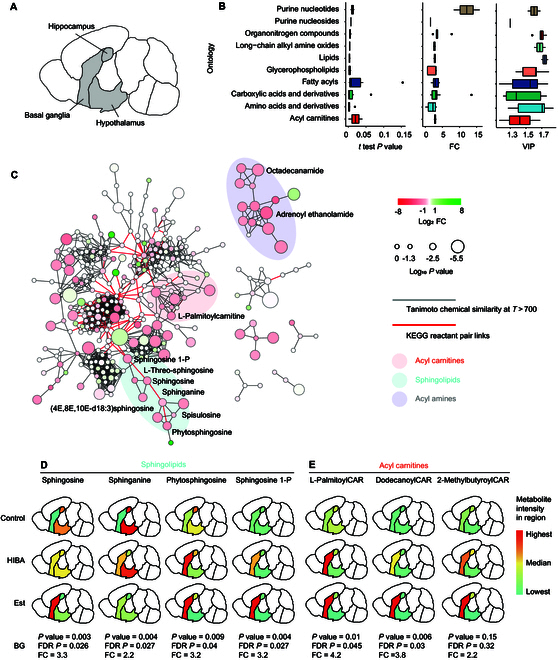
The mouse brain heat map visualizing the metabolites in the brain. (A) Brain heat map model for better understanding of the 3 brain regions (hippocampus, hypothalamus, and BG) of interest in this experiment. (B) Boxplot of FC, *P* value, and VIP value of 49 metabolites divided into 10 categories in BG that are significantly altered by HIBA. (C) MetaMapp visualization of metabolomic data highlighting the differential metabolic regulation in BG. Red edges denote KEGG reactant pair links; gray edges symbolize Tanimoto chemical similarity at *T* > 700; unknowns are left out of these graphs for visual clarity. Metabolites found significantly up-regulated under exposure to HIBA (*P* < 0.05) are related to node sizes. Node color reflects FC. Metabolites that were not found to be differentially regulated were left unlabeled for visual clarity. Three major network clusters were labeled with class name. (D and E) The brain heat map was used to display the screened 7 representative metabolites in BG. Colors are annotated according to the peak intensities of metabolites in BG, with red for high concentrations and green for low concentrations.

To better illustrate the quantitative results of brain metabolism, a visual heat map of brain metabolome was constructed, including hippocampus, hypothalamus, and BG (Fig. 4A). Forty-five significantly changed metabolites [*P* < 0.05, variable influence on projection (VIP) > 1, FC > 1.5 or < 0.6] in BG between the control and HIBA-treated group were selected as representative metabolites. As shown in Fig. 4B, these 45 metabolites can be classified into 10 categories (Data S7). Changes in the BG metabolic network were globally visualized in MetaMapp [19] graphs as shown Fig. 4C. The representative significantly altered metabolites of acyl carnitines, sphingolipids, and acyl amines were displayed, while not labeling the unchanged compounds. The edge and node information of the BG metabolic network could be obtained in Data S8. The curated metabolites were clustered into 8 major network clusters. The amines and related derivatives are mainly involved in acyl carnitines, sphingolipids, and acyl amines. In order to better represent the expression levels of metabolites in categories of acyl carnitines and sphingolipids in BG, we show them by brain heat map. Four representative sphingolipids, including sphingosine, sphingosine 1-phosphate, phytosphingosine, and sphinganine, were displayed, with false discovery rate *P* value lower than 0.05 and FC value over 2, as shown in Fig. 4D. It is interesting to note that acetyl-l-carnitine (ALCAR) showed significant up-regulation in both the HIBA and Est groups. This suggests that the expression level of ALCAR may be related to sleep, as ALCAR can target brain metabolism, enhance cellular energy, and act as a powerful neuroprotective agent. The 3 most representative ALCAR were l-arachidonoylcarnitine, O-dodecanoylcarnitine, and 2-methylbutyroylcarnitine and were visualized in Fig. 4E. The extracted ion chromatogram (EIC) of aligned spot of sphingolipids and ALCAR metabolites in mouse brain treated with HIBA was displayed in Fig. S3. The LC-MSMS spectrum could be obtained in Fig. S4. The brain heat map of other representative metabolites in the brain was displayed in Fig. S5. Based on the analysis of representative metabolites, it is discernible that the Est group exhibited a considerable up-regulation in the screening. One possible explanation is that HIBA and Est may share similar molecular pathways in promoting sleep.

### Gut microbiota community and intermediary effect

**Fig. 5.**
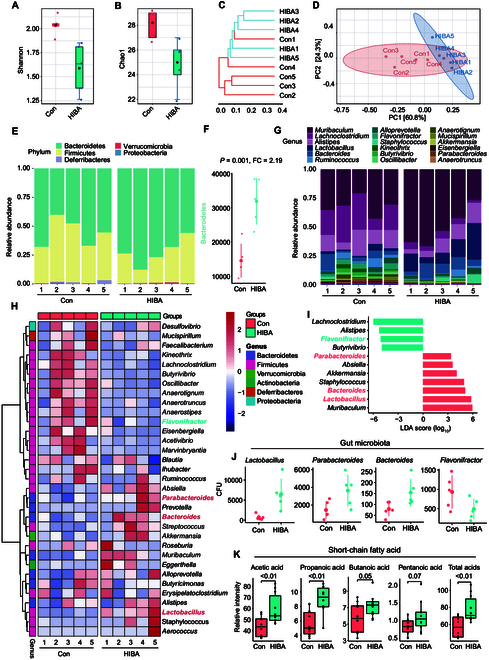
Effects of HIBA on microbiota in mice. The gut microbiota community analysis of HIBA mice (*n* = 5) and control (Con) mice (*n* = 5). Alpha diversity: (A) Shannon and (B) Chao1. (C) Cluster analysis. (D) Beta diversity. Changes and difference in microbiota on (E) phylum level and (G) genus level. (F) Stripchart plot of *Bacteroides*. Error bars represent the SD, and the mean value is represented by a larger dot. (H) Heat map of microbiota of genus level. (I) LDA analysis. (J) Stripchart plot of the microbiotas of the top 4 microbiotas of genus level. Error bars represent the SD, and the mean value is represented by a larger dot. (K) Boxplot of SCFAs. Data are expressed as mean values ± SEM. *P* values were calculated using Student’s two-tailed *t* test.

**Fig. 6.**
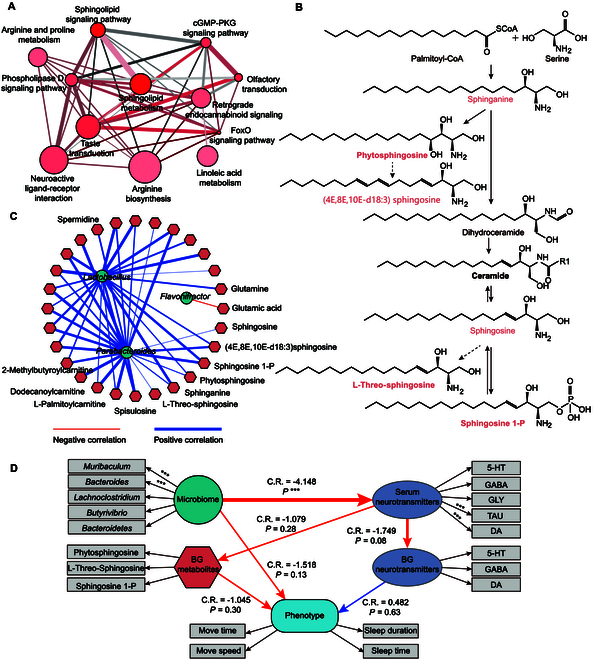
Analysis of pathways and intermediary effect. (A) Quantitative metabolite set enrichment analysis (qMSEA) based on 38 metabolites with KEGG ID through ConsensusPathDB web tool, functional module over representation analysis. Node size: metabolite numbers, node color: *P* value, edge width: shared metabolites%, edge color: metabolites from input. (B) Sphingolipid biosynthesis pathway. The sphingolipids detected and significantly increased in HIBA-treated mice’s BG were labeled in orange. (C) Correlation network between microbiome and BG metabolites. Blue line, negative correlation; red line, positive correlation. The linewidth was related to *P* value. (D) Structural equation modeling for analysis of intermediary effect between microbiome, serum neurotransmitters, BG neurotransmitters, BG metabolites, and phenotype. Red line, negative correlation; blue line, positive correlation. C.R. for correlation value is represented by the thickness of the mapping line. ***P < 0.001.

In the above analysis of neurotransmitters, we find that there are significant changes in 6 common neurotransmitters in the blood in the HIBA-treated group, and then in different regions of the brain. The gut–brain axis is an important pathway that connects the brain and the gut, and neurotransmitters play a crucial role in transmitting signals within this axis. Based on the experimental results of the aforementioned neurotransmitters, we aim to investigate the molecular mechanisms of HIBA’s sleep-promoting effects through the gut–brain axis. To reveal whether HIBA affected microbiota diversity, we tested the alpha diversity of the HIBA group and control group samples (Figs. 5A and 6B). Chao1 and Shannon index represented the total number of species, which confirmed that the diversity and uniformity of the HIBA-treated group was lower than that of the control group. Cluster analysis was utilized to study the effects of Control and HIBA groups on mice fecal bacteria (Fig. 5C). There were differences in the fecal bacteria between the HIBA and control groups of mice, but they were not completely distinguishable under the cluster analysis method. As shown in Fig. 5D, the beta diversity, as PCA, indicated that the control group and HIBA group were markedly different, further suggesting that HIBA had effects on the composition of microbiota community. The gut microbiota comprises a range of dominant phyla, among which *Bacteroides*, *Firmicutes*, and *Verrucomicrobia* are commonly represented. As depicted in Fig. 5E, alterations in the relative abundance of various phyla, namely, *Bacteroidetes*, *Firmicutes*, *Deferribacteres*, *Verrucomicrobia*, and *Proteobacteria*, were observed. Notably, compared to the control group, the intervention with HIBA led to a significant increase in the relative abundance of *Bacteroidetes* (*P* = 0.001, FC = 2.19) (Fig. 5F).

To ascertain the particular genus responsible for metabolizing HIBA in mice colon, we conducted an analysis of the relative abundance of genus scores, as depicted in Fig. 5G. Compared with the control group, the relative abundance of *Anaerostipes*, *Anaerotignum*, *Marvinbryantia*, *Butyrivibrio*, *Acetivibrio*, *Eisenbergiella*, *Kineothrix*, *Oscillibacter*, *Lachnoclostridium*, *Anaerotruncus*, and *Flavonifractor* in the HIBA group decreased, while that of *Parabacteroides*, *Bacteroides*, *Absiella*, *Muribaculum*, *Streptococcus*, *Akkermansia*, and *Lactobacillus* increased. Heat map (Fig. 5H) was used to better show the genus of differences (Data S9). Bacterial genus differentially represented between the control and HIBA groups identified by linear discriminant analysis (LDA) effect size (Data S10). Four commonly observed and abundantly present genera (*Flavonifractor*, *Parabacteroides*, *Bacteroides*, and *Lactobacillus*) are highlighted (Fig. 5I). The stripchart is used to show that the abundance of the 4 genera differs significantly (Fig. 5J). Studies have found that short-chain fatty acids (SCFAs) play a key role in the transmission of messages between gut microbes and the brain, and are closely related to many neurological diseases [20].The butyrate produced by specific microorganisms mainly exists in the colon. After being absorbed and utilized by the host, it affects a variety of host physiological processes through specific transporters or receptors, and exerts neuropharmacological effects. In the HIBA group, acetic acid (*P* < 0.01), propionic acid (*P* < 0.01), butanoic acid (*P* = 0.05), and pentanoic acid (*P* = 0.05) increased significantly (Fig. 5K and Data S11).

The results obtained from the quantitative metabolite set enrichment analysis (qMSEA) analysis demonstrated that BG had significantly enriched pathways (adjusted *P* < 0.05), as depicted in Fig. 6A. Among the top enriched BG pathways were those ranging from sphingolipid metabolism to linoleic acid metabolism, which are presented in Data S12. Notably, sphingosine, an important molecule used by cells to synthesize ceramides, can be derived from the combination of palmitoyl-CoA (coenzyme A) and serine. Interestingly, the genomes of *Bacteroides* spp. and their related bacteria encode serine palmitoyltransferase (SPT), which allows them to produce sphingolipids, thus impacting the levels of ceramides in the host metabolic pathways [21]. In the sphingolipid biosynthesis pathway (Fig. 6B), the 6 sphingolipids with *P* value lower than 0.05 and FC over 2 were labeled in orange. The sphingolipids (4E,8E,10E-d18:3) sphingosine and l-threo-sphingosine are not included in the pathway. However, they include sphingolipids, so the dashed line was used to connect them to the metabolites in pathways. To better understand the relationship between that microbiome with metabolites in BG, the Spearman correlation value was calculated by R packages, and the network was visualized by cytoscape (Data S13). With filter tolerance to 0.7 and −0.7, the simplified network was generated, as shown in Fig. 6C. It could be noticed that *Flavonifractor*, *Parabacteroides*, and *Lactobacillus* survived. Sphingolipids and ALCAR have negative relation with *Parabacteroides.* PICRUSt2 analysis was used to predict metabolic functions (Data S14). A structural equation modeling was developed to uncover the direct and indirect contributions of HIBA to the microbial communities, metabolites, and phenotype (Fig. 6D). The microbiome could strongly impact on the serum neurotransmitters, with C.R. = −4.148 and *P* < 0.001, while the serum neurotransmitters will impact the BG neurotransmitters, with C.R. = −1.747 and *P* < 0.08. BG neurotransmitters have positive impact on phenotype, including move time, move speed, sleep time, and sleep duration, while the microbiome and metabolites in BG have negative impact on phenotype. The serum neurotransmitters, TAU and DA, and the microbiome, *Muribaculum* and *Bacteroides*, contributed most. All these information of structural equation modeling could be obtained in Data S15.

## Discussion

Through multiple animal sleep and sedation experiments, combined with the separation and purification of ODS-A, ODS-AQ, ODS-D, and other chromatographic columns, we have identified HIBA from walnut DJF for the first time. By analyzing 8 neurotransmitters in 3 key parts of the brain related to sleep (hypothalamus, BG, and hippocampus), we found that ACH, GLY, and GABA levels in 3 brain regions were up-regulated. ACH is a neurotransmitter that can be used as a selective agonist of cholinergic receptors, ubiquitous in the peripheral and central nervous system [22], which can affect the memory storage and cognitive functions in the cortex and subcortical circuits [23], affecting behavioral activities, sleep–wake, learning, and memory. In the HIBA-treated BG, the increase of ACH indicated that HIBA might stimulate the expression of ACH in the brain to promote sleep. GLU can be converted into GAM (L-glutamine) or GABA, and vice versa, GLU can also be formed from GAM or GABA. GABA produces a similar sedative inhibitory effect by activating neurons and receptors in the brain. In the brain, we can observe a significant up-regulation of GABA. Previous studies have confirmed that supplementation with GABA-rich beverages can alleviate anxiety in mice, improve sleep [24], and increase the levels of inhibitory transmitters GABA and GLY in the brain. The increased concentration of GABA and GLY in BG indicates that HIBA could regulate the levels of inhibitory transmitters GABA and GLY to promote sleep. 5-HT is a precursor of melatonin, which is a hormone that regulates biological rhythms and is often used to treat sleep disorders. The increase of 5-HT in the brain will cause the body to feel tired and sleepy, and it will be easier to sleep. The significant up-regulation of 5-HT in the HIBA group indicated that HIBA may improve the sleep of mice by adjusting the level of 5-HT in hypothalamus. DA is mainly distributed in the hypothalamus and participates in sleep regulation, endocrine, and body movements. During the awakening period, DA neurons increase pulsed firing and increase DA release in related parts of the forebrain [25]. In serum, DA sharply decreased in the HIBA group. Unfortunately, there is no increase or decrease in DA levels in the brain tissue. As one of the highest contents of endogenous amino acids in the central nervous system, TAU is mainly found in the excitable tissues such as the brain. TAU modulates neurotransmission by triggering the transmission of inhibitory neurons of GABAA and GLY receptors, and enters cells through sodium-dependent taurine transporters [26]. In BG of the HIBA-treated mice, GABA, GLY, ACH, and TAU, with varying degrees of significant up-regulation. It is hypothesized that HIBA exerts sedative and hypnotic effects by modulating neurotransmitter levels in both the serum and brain of mice, particularly acting on 5-HT and GABAergic neurons, ultimately impacting the sleep–wake cycle. It is speculated that HIBA may exhibit GABA-like effects, either through conversion to GABA within the gastrointestinal tract or within the body.

In addition to its effects on neurotransmission, HIBA also exerts an impact on brain metabolism. Metabolomic investigation revealed that HIBA predominantly modulates the metabolism of BG by up-regulating and down-regulating the levels of 83 and 6 metabolites, respectively. The modulated metabolites belong chiefly to amine and related derivatives, acyl carnitines, sphingolipids, and acyl amines. Notably, the sphingolipid biosynthesis pathway was found to be notably enriched in HIBA-treated mice, supported by the significant increase in 6 sphingolipids within BG. Sphingolipid catabolism is required for adult sleep behavior [27], and there are significant expression differences of sphingolipids between sleep and wakefulness states [28]. Sphingosine, which arises from the condensation between palmitoyl-CoA and serine, plays a crucial role as a fundamental precursor for ceramide biosynthesis. Ceramides, composed of sphingosine and very long-chain fatty acids, form the structural backbone of all sphingolipids [29]. Ceramide could preferentially influenced microglia in the thalamic reticular nucleus (TRN), and local depletion of TRN microglia produced similar impaired wakefulness [30]. Another molecule worth mentioning is ALCARs. It previously reported sleep-induced elevation of acyl-carnitines, demonstrating the relevance of lipid conjugation of small metabolites in brain regional circadian alterations [31]. HIBA up-regulated ALCARs, such as palmitoylcarnitine, l-arachidonoylcarnitine, oleoyl-l-carnitine, and O-dodecanoylcarnitine. Increasing ALCAR brings the benefits straight to the brain and potentially improves the quality and length of sleep [32]. Oleoyl-carnitine has been shown to exert palliative activity by binding to the glycine transporter GlyT2, thereby promoting the beneficial role of sleep in brain function via similar mechanisms [33]. Furthermore, myristoylcarnitine, and palmitoylcarnitine were significantly changed in sleep fragmentation, also including adenosine monophosphate (AMP), hypoxanthine, l-glutamate, l-aspartate, l-methionine, and glycerophosphocholine, involving the alanine, aspartate, and glutamate metabolism pathway [34]. In the following correlation study, it was found that *Lactobacillus* and *Parabacteroides* had a strong positive correlation with these 4 ALCARs (Spearman’s correlation coefficient *P* > 0.7). In addition to this, other significantly up-regulated metabolites include purine nucleosides, endocannabinoids, and glycerophosphocholine. As reported, the AMP concentration increased during sleep deprivation but normalized during subsequent recovery. Furthermore, the nucleotides adenosine-5′-P and uridine-5′-P act as the sleep promoters. The intracellular AMP/ATP (adenosine triphosphate) ratio controls the activity of AMP-activated protein kinase, which is a potent energy regulator and is recently reported to play a role in the regulation of sleep homeostasis [35]. Also, *Lactobacillus* (*P* > 0.7) and *Parabacteroides* (*P* > 0.9) had a strong positive correlation with AMP.

With the increasing understanding of the intestinal–brain axis, the 2-way communication channel connects the brain and intestines; however, the potential therapeutic role of gut microbiota modulation to improve sleep is unexplored. The activation of the hypothalamus–pituitary–adrenal (HPA) axis has been found to exert a significant impact on the gut microbiota composition. Notably, a bidirectional signaling communication channel between the gut microbiota and the HPA axis has been identified, indicating a complex relationship [36]. Moreover, the administration of probiotics to mice has been demonstrated to decrease the basal activity of the hypothalamic–pituitary–adrenal (HPA) axis and the levels of corticosterone induced by stress [37]. Various microbial activities influence the HPA axis through nerve signaling, SCFAs, and epigenetic mechanisms. Evidence suggests that SCFAs, the main byproducts of fiber fermentation in the gut, may affect sleep via gut–brain communications [38]. Injecting butyrate, an SCFA, into mice has shown a 70% increase in non-REM sleep [39]. This highlights the importance of microbial molecules in sleep regulation and their potential use in developing new therapies for sleep disorders [40]. The sodium acetate and sodium butyrate could induce marked changes to clock gene Bmal1 and Per2 expression in hepatic cells of mice [41]. GABA can improve the sleep of mice and increase the total content of SCFAs, especially butyric acid. HIBA, with a structure similar to GABA, may play a similar role, affecting the total content of SCFAs, then indirectly affecting the brain by regulating the immune system and vagus nerve activity.

The gut microbiota is a complex network of various microorganisms that exhibit rhythmic oscillation. Certain groups of bacteria, including *Clostridiales*, *Lactobacillales*, and *Bacteroidales*, such as *Bacteroidetes*, *Firmicutes*, *Ruminococcaceae* spp, *Clostridia* spp, *Lachnospiraceae* spp, *Oscillospira*, *S24-7* spp, Anaeroplasma, *Bacteroides*, and *Lactobacillaceae* spp, were observed to follow a 24-hour oscillation pattern in mice. Additionally, a preliminary study demonstrated that individuals with superior sleep quality had a higher abundance of *Blautia* and *Ruminococcus* (Firmicutes) and a lower proportion of *Prevotella* (Bacteroidetes), indicating a positive correlation between sleep quality and the *Firmicutes*/*Bacteroidetes* ratio [42]. Several microbiota manipulations, in particular some probiotics, are known to be beneficial for promoting sleep quality, especially the most studied *Lactobacillus* and *Bifidobacteria*. *Parabacteroides distasonis* associate with facilitated sleep/clock realignment after chronic disruption of rhythms [43]. Adverse changes in sleep resulting from sleep fragmentation are associated with reduced levels of Parabacteroides in the gut microbiome during post-sleep fragmentation [44]. This means that the abundance of *Parabacteroides* is closely related to the quality of sleep. *Flavonifractor* is positively correlated with fatigue in patients with major depressive disorder, including mood disorders and daytime sleepiness, which means that *Flavonifractor* is a kind of microflora that causes mood and sleep abnormalities, and controlling its composition ratio in the gut will benefit mood and sleep [45]. When “sleep quality” improved, the gut microbiota was influenced by gut–brain connectivity, manifested as an increase in *Bacteroides*; conversely, the abundance of *Bacteroides* was positively correlated with sleep quality [46]. Our results found that HIBA mainly up-regulated *Parabacteroides*, *Bacteroides*, and *Lactobacillus* and down-regulated *Flavonifractor*, affecting glutamate and asparagine biosynthesis and nucleic acid processing based on 16*S* rRNA marker gene PICRUSt analysis, and glutathione, pyrimidine, and phospholipid metabolism based on significantly changed metabolites in BG. These results are enough to prove that HIBA can indeed change the microbiota of mice, but whether these changes at the level of predominant phylum or genus are directly related to the promotion of sleep by HIBA will be further studied later. In addition, it is worth mentioning that the sterile mouse experiments will increase whether HIBA affects brain metabolism and sleep quality in mice through the gut flora, and this one is also a limitation of this study.

The structural equation modeling uncovered the direct and indirect contributions of the microbial communities and metabolites to phenotype. HIBA, with a molecule structure similar to GABA, could be metalized by microbiome (*Muribaculum*, *Bacteroides*, etc.) and regulated the serum neurotransmitters (TAU, DA, etc.). The serum neurotransmitters affect the neurotransmitters and metabolites in BG, possibly through the intestinal–brain axis. Finally, the neurotransmitters and metabolites in BG regulated the mouse phenotype, including move time/speed and sleep time/duration. Furthermore, the microbiome also has a direct effect on sleep and activity behavior.

To sum up, we discovered a GABA-like molecule, HIBA, from DJF and explored the molecular mechanism of HIBA’s sedative and hypnotic action with brain metabolism, blood metabolism, and fecal microbiota. HIBA up-regulated *Parabacteroides*, *Bacteroides*, and *Lactobacillus*, promoted the level of SCFA in the intestine, and interfered with sphingolipids, ALCARs, and purine nucleoside metabolism in BG. Although the underlying molecular mechanisms remain inadequately understood, HIBA has the potential to be used in sedative and hypnotic drug development.

## Materials and Methods

### Crude separation

Weigh 10 kg of DJF, pass through a 40-mesh sieve after crushing, and place it in a 100 L extraction tank. Placed it in a 100 L extraction tank, add 70% ethanol according to 1:10 (kg:L) material-liquid mass-volume ratio. Stir and extract for 4 h at 60 °C, filter, and take the filtrate. The filter residue is extracted again with alcohol according to the above method, and the filtrate is collected and combined with the first filtrate. Concentrate under reduced pressure to an appropriate volume, which is the ethanol extract of DJF, and store it frozen at −20 °C. The filter residue after alcohol extraction was extracted twice with water, and the filtrate was collected and concentrated, which is the water extract of DJF. Take an appropriate amount of the water extract of DJF, add 3 times the volume of absolute ethanol, and keep stirring for 3 h, and let it stand overnight. Then, the water extract (A) is dried to obtain solid particles and stored at −20 °C. Take an appropriate amount of the mixed extract of the DJF on the AB-8 type macroporous resin column (10 cm × 150 cm), followed by gradient elution with water and 30%, 50%, and 70% ethanol), and the eluent was combined according to the TLC result. According to the TLC test results, the eluents with the same composition are combined, and the eluent is divided into 3 parts: B (water elution), C (30% and 50% elution), and D (70% elution). After being concentrated under reduced pressure, they were frozen and stored at −20 °C.

### Sedative and hypnotic effects

Sixty SPF (specific pathogen-free) male ICR (Institute of Cancer Research) mice, aged 4 weeks, were adaptively reared for 1 week. They were then randomly divided into 6 groups, each consisting of 10 mice. The groups were as follows: the blank group, the test group receiving intragastric solvent, groups A, B, C, and D receiving a dose of 1.0 g/kg body weight, and the positive control group receiving 2 mg/kg body weight of Est. After 14 days of continuous gavage, the experiment commenced. The test is carried out in a quiet environment around 25 °C.

An open field maze of 4 units (each unit 50 cm × 50 cm × 38 cm) consisting of 4 activity spaces was used to conduct the experiment, and 4 mice were detected each time. Clean the open field with alcohol before the experiment, and start the experiment 30 min after the sample group is administered (15 min for the positive control group). Gently grasp the tail of the mice during the experiment, place the mice in the middle of the open-field maze of each unit, let the mice adapt to the maze for 5 min, then click the software start button to activate the software and track the mice’s movement path. Record the average movement speed, movement time and stay time of the central area of the mice within 10 min. Use EthoVision XT 11 software for data analysis. At the end of each test, clean up urine and feces, and spray 5% to 10% ethanol on the bottom and inner walls of the labyrinth, and wipe them clean with paper towels. After thoroughly eliminating the odor left by the previous group of mice, the next group of mice was tested to ensure that the odor and residue did not influence the results of the subsequent experiments.

Direct sleep observation was performed the next night after the open field experiment. Thirty minutes after the gastric administration, surveillance cameras were used to observe and record the sleep time of the mice in each group within 12 h after the gastric administration. Before the formal experiment, a preliminary experiment was carried out to determine the intraperitoneal injection dose of pentobarbital sodium. After intraperitoneal injection, all mice fell asleep and the duration of sleep was moderate as the standard, and this dose (50 mg/kg body weight) was used for formal experiments. Mice in each group were given intraperitoneal injection of sodium pentobarbital to induce sleep after intragastric administration for 30 min. The sleep latency of mice was recorded as the time from pentobarbital sodium injection to the disappearance of the righting reflex. The time from the disappearance of the righting reflex to the reappearance of the righting reflex was recorded as the sleep time of the mice.

### Separation of active components

Part B (water elution), with sedative and hypnotic function, was loaded onto the MCI column (5 cm × 100 cm), followed by gradient elution with water and 10%, 30%, and 50% ethanol at a flow rate of 15 ml/min. The automatic collector collects the eluate in each tube of 20 ml, uses the TCL method to track and monitor the components in the eluate, and combines the same components according to the Rf value (received signal strength indicator) and the result of the color reaction to obtain B-1 (water elution), B-2 (10% and 30% elution), and B-3 (50% elution) 3 components. Mouse experiments were conducted to analyze the sedative and hypnotic effects of the B-1, B-2, and B-3 components, which were separated using the MCI column, and the sedative and hypnotic effects were analyzed in each component. Then, the objective was to isolate and structurally identify a specific component exhibiting sedative and hypnotic activity. The B-1 component with sedative and hypnotic function was repeatedly loaded onto the ODS chromatographic column until 4 monomer components were obtained, recorded as compounds 1, 2, 3, and 4. To determine the structure, the compound was dissolved in either heavy water (D_2_O) or deuterated dimethyl sulfoxide (DMSO-_d6_), and its spectrum data, including ^1^H NMR and ^13^C NMR, were thoroughly analyzed.

The monomer compounds were tested on animals according to the method 2.2 to determine their sedative and hypnotic effects. Briefly, a total of 60 SPF male ICR mice aged 4 weeks (body weight around 18 g) were purchased, and after adaptive feeding for 7 days, they were randomly divided into groups. They are the blank control group, the experimental group (compounds 1, 2, 3, and 4), and the positive control group (Est), each with 10 animals. The blank group and the experimental group were intragastrically administered at a dose of 0.5 g/kg body weight, and the positive control group was intragastrically administered at a dose of 2.0 mg/kg body weight. The stomach was gavaged for 14 consecutive days at the same time, and the experiment was started in a quiet environment at around 25 °C.

### Sample preparation

Twenty-four hours after the last behavioral experiment, blood was taken from mice to obtain serum. Then, the mice were perfused into the heart with 4 °C precooled normal saline. Quickly peel off the mice hypothalamus and left and right hippocampus on the ice box, and store them at −80 °C for later use. Serum: Aspirate 100 μl of serum into a 1.5 ml EP tube, add 300 μl of extractant (acetonitrile) and vortex for 30 s, then ultrasonicate in an ice bath for 5 min, centrifuge at 12,000 rpm for 15 min at 4 °C, and take the supernatant. Dry in a vacuum freeze dryer, reconstitute with 150 μl of resolvent (acetonitrile: water= 1:1, v/v), vortex for 30 s, sonicate in an ice bath for 5 min, centrifuge for 15 min, and take the supernatant in a chromatographic bottle, to be tested. Tissue: First, cut 10 mg of brain tissue sample on dry ice, place it in a 1.5 ml EP tube, and add 300 μl of extractant (acetonitrile: isopropanol: water = 3:3:2, v/v/v). Homogenize extraction with 2 stainless steel beads with a diameter of 3 mm. Then take it out for 5-min ultrasonic extraction in an ice bath, and finally centrifuge at 12,000 rpm for 15 min at 4 °C to take the supernatant. Dry in a vacuum freeze dryer, add 150 μl of resolvent for reconstitution, vortex for 30 s, sonicate in an ice bath for 5 min, centrifuge for 15 min, and take the supernatant in a chromatographic bottle for testing.

### Neurotransmitter analysis

The analysis of neurotransmitters was mainly based on published methods [47] and further optimized with appropriate parameter adjustments. Briefly, the Agilent Infinity Poioshell 120 HILIC-Z (2.1 mm × 100 mm, 2.7 μm) column was used, and the column temperature was kept at 35 °C. The injection volume is 2 μl. The flow rate is 0.25 ml/min. Chromatographic conditions: mobile phase A is 20 mM ammonium formate aqueous solution (pH 3), and mobile phase B is acetonitrile–water solution (v/v, 9:1) containing 20 mM ammonium formate. The gradient elution conditions are as follows: 0 to 15 min, 5% A; 15 to 17 min, 35% A; 17 to 18 min, 1% A. The total running time is 18 min. Mass spectrometry conditions: ESI positive ion mode, multiple reaction monitoring mode (MRM), capillary voltage 5.5 kV. Eight kinds of neurotransmitter mass spectrometry detection parameters are shown in Table [48].

**Table.** MRM parameters for eight neurotransmitters.

Analyte	Q1 mass (Da)	Q2 mass (Da)	Dwell (ms)	DP (V)	EP (V)	CE (eV)	CXP (V)
Ach	146	42.9	50	75	10	37	13
5-HT	177	160	50	60	10	17	13
DA	154.3	137.1	50	60	10	14.5	13
GABA	104	87	50	60	10	15	13
Glu	148	130.1	50	40	10	12	13
Asp	134	74	50	60	10	15	13
Gly	76	30	15	60	10	15	13
Tau	126	108	15	60	10	12	13

### SCFA determination

The extraction of SCFAs was improved based on previously published methods [49]. The SCFAs were analyzed using gas chromatography (GC)–MS method and GCMS-QP2010 (Shimadzu Corporation, Japan). The column model is DB-Wax UI, 30 m × 0.25 mm × 0.25 μm (Thermo, USA). The sampler parameter settings are as follows: temperature, 250 °C; spectrum ratio, 10:1; carrier mode, constant current; and value, 1 ml/min. The instrument is initially set at a temperature of 60 °C, with a heating rate of 15 °C per minute until it reaches 240 °C. It is then maintained at this temperature for 10 min. The ionization mode is EI, the emission current is 1 mA, and the electron energy is 70 eV. The interface temperature is 250 °C, the source temperature is 210 °C, and the voltage is 2,000 V.

### 16*S* rRNA

A genomic DNA kit (Omega Bio-tek, GA, USA) was used to extract DNA from the bacterial flora in feces components. The bacterial 16*S* rRNA sequencing gene (V3–V4 region) was amplified from the entire genome by primer pairs. The amplicons are quantified, combined, and sequenced by the Illumina MiSeq machine. The barcode and connector sequence in the original sequence of the machine have been removed. QIIME 2 was used to splice double-ended sequences. Finally, sequences with 97% similarity are classified as OUT. Gene function is predicted by PICRUSt2 software and analyzed by the Kyoto Encyclopedia of Genes and Genome (KEGG) database.

### Nontarget metabolomics analysis

To obtain a comprehensive metabolic profile in serum and brain, an Acquity UPLC (ultra-performance liquid chromatography) system (Waters Corporation, Milford, MA, USA) was connected to a Thermo Orbitrap QE Focus mass spectrometer (Thermo Fisher Scientific Inc., Waltham, MA, USA) equipped with electrospray ionization (ESI) source. The chromatographic separation was performed in an Acquity UPLC system equipped with Acquity UPLC HSS T3 (150 × 2.1 mm, 1.8 μm, Waters). As previously reported, gradient elution of analytes was performed [50].

### UPLC-MS data statistics

The UPLC-MS raw data files were converted to ABF format using ABF converter (available at https://www.reifycs.com/AbfConverter/). The deconvolution, peak picking, alignment, and identification of compounds were performed using MS-DIAL ver. 4.00 software. The precise parameter settings for this process were as follows: MS1 tolerance of 0.005 Da; MS2 tolerance of 0.01 Da; a minimum peak height of 20,000 amplitude; mass slice width of 0.1 Da; linear weighted moving average smoothing method with a smoothing level of 5 scans; and a minimum peak width of 10 scans. For positive mode, adduct ion settings included [M + H]^+^, [M + NH_4_]^+^, [M + Na]^+^, [2M + H]^+^, [2M + NH_4_]^+^, and [2 M + Na]^+^, while for negative mode, adduct ion settings included [M–H]^–^, [M + Cl]^–^, [M + FA-H]^–^, and [2M-H]^–^. Compound annotation was performed by aligning retention times, accurate precursor masses, and MS/MS spectra with those in the MoNA library (accessible at https://mona.fiehnlab.ucdavis.edu/). Missing data were substituted with ^1^/_10_th of the minimum value (default value, 100).

### Statistical analysis

Statistical analysis was performed by log transformation and Pareto scaling. PCA was used for multivariate statistics and visualization, specifically for outlier detection. All results are shown as the mean ± standard deviation (SD). Analysis of variance (ANOVA) and Duncan’s test for multiple comparisons were used to analyze data. The visualization of metabolites is performed using the R package, ggplot, ggpubr, pheatmap, and ropls [51]. Microbiological analyses were performed using the statistical software R. Spearman rank correlation analyses and FC calculations were conducted using R. Structural equation modeling analysis was performed using software R (mediation), based on nonsignificant chi-square test.

## Data Availability

The authors declare that the data supporting the findings of this study are available within the paper and its supplementary information files. These data are available at Metabolight (https://www.ebi.ac.uk/metabolights/), where they have been assigned Project ID MTBLS5904. Code availability: R codes and associated data for reproducing part of the figures in this study have been deposited at GitHub (https://github.com/JianJi2016/HIBA_brain). Unless noted otherwise, all other analyses including MetaMapp were done using web-based portal and visualized in standalone GUI software such as Adobe Illustrator (version 25.0) and CytoScape (version 2.7.2).
