# Erratum to “Sleep Promotion by 3-Hydroxy-4-iminobutyric Acid in Walnut *Diaphragma Juglandis Fructus*”

**DOI:** 10.34133/research.0585

**Published:** 2025-01-27

**Authors:** Jian Ji, Yongli Ye, Lina Sheng, Jiadi Sun, Qianqian Hong, Chang Liu, Jun Ding, Shuxiang Geng, Deping Xu, Yinzhi Zhang, Xiulan Sun

**Affiliations:** ^1^ State Key Laboratory of Food Science and Technology, School of Food Science and Technology, National Engineering Research Center for Functional Food, Synergetic Innovation Center of Food Safety and Quality Control, Jiangnan University, Lihu Avenue 1800, Wuxi, Jiangsu 214100, P.R. China.; ^2^ Department of Chemistry, Wuhan University, Wuhan, Hubei 430072, PR China.; ^3^ Yunnan Academy of Forestry and Grassland, Kunming, Yunnan 650201, PR China.; ^4^ College of Food Science and Pharmacy, Xinjiang Agricultural University, No. 311 Nongda Dong Road, Ürümqi, Xinjiang Uygur Autonomous Region 830052, P.R. China.

In the Research Article “Sleep Promotion by 3-Hydroxy-4-Iminobutyric Acid in Walnut *Diaphragma juglandis Fructus*,” [1] the authors have identified an error in the Results section and Figure 1. The original statement was:

“C2 was annotated as 3-hydroxy-4-iminobutyric acid (HIBA) (Fig. 1N). The structure of HIBA is similar to that of GABA, except that HIBA only has one more -OH than GABA (Fig. 1O), which indicates that HIBA may have a similar molecular function to GABA.”

**Figure:**
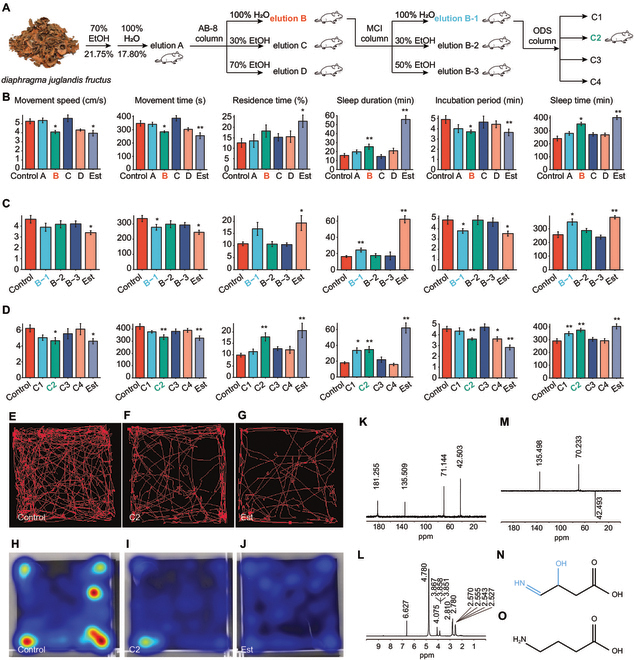


This has been corrected as follows:

“C2 was annotated as 3-hydroxy-4-iminobutyric acid (HIBA) (Fig. 1N). The structure of HIBA is similar to that of GABA, except that HIBA has one more -OH group compared to GABA. The carbon-4 position of HIBA is connected to an imino group (-NH=), while the carbon-4 position of GABA is connected to an amino group (-NH_2_) (Fig. 1O). It indicates that HIBA may have a similar molecular function to GABA.”

The panels of Figure 1 have been corrected in the original publication and below (Fig. 1).
