# Supplementary material for: Sleep Promotion by 3-Hydroxy-4-Iminobutyric Acid in Walnut Diaphragma juglandis Fructus
Source: Research (Wash D C). 2023 Sep 19;6:0216. doi: 10.34133/research.0216 (PMC10508226; doi:10.34133/research.0216)
Supplement: Supplementary 1 — Figs. S1 to S25. Tables S1 to S6 Data S1 to S15 [file research.0216.f1.zip › Supplementary Information.pdf]

Supplementary Information

Molecular mechanisms of mouse sleep promotion induced by HIBA extracted from *Diaphragma Juglandis Fructus*

Jian Ji[1,4†], Yongli Ye[1†], Lina Sheng[1], Jiadi Sun[1], Qianqian Hong[1], Chang Liu[1], Jun Ding[2], Shuxiang Geng[3], Deping Xu[1], Yinzhi Zhang[1], and Xiulan Sun [1*]

[1] State Key Laboratory of Food Science and Technology, School of Food Science and Technology, National Engineering Research Center for Functional Food, Synergetic Innovation Center of Food Safety and Quality Control, Jiangnan University, Lihu Avenue 1800, Wuxi, Jiangsu 214100, P.R. China.

[2] Department of Chemistry, Wuhan University, Wuhan, Hubei 430072, PR China

[3] Yunnan Academy of Forestry and Grassland, Kunming, Yunnan 650201, PR China

[4] College of Food Science and Pharmacy, Xinjiang Agricultural University, No. 311 Nongda Dong Road, Ürümqi, Xinjiang Uygur Autonomous Region 830052, P.R. China.

[†]These authors contributed equally to this work.

[*] Address correspondence to: Xiulan Sun, sxlzyz@jiangnan.edu.cn.

Figures list

Figure 1 The optimized extraction parameters of active ingredient of *diaphragma juglandis fructus*. **a** The influence of material-to-liquid ratio on the yield of crude extract. **b** The influence of extraction time on the yield of crude extract. **c** The influence of extraction temperature on the yield of crude extract. **d** The influence of ethanol concentration on the yield of crude extract. **e** The influence of number of extractions on the yield of crude extract

Figure 2 The TLC diagram of eluted components of MCI column and monomer compounds. **a** Three eluted components of MCI column. **b** Four monomer compounds.

Figure 3 The EIC of aligned spot of BG metabolites in mice brain treated with HIBA (n = 8) and Est (n = 8). **a** The EIC of aligned spot Sphingosine like metabolites, sphingosine, (4E,8E,10E-d18:3)sphingosine, sphingosine 1-phosphate, Phytosphingosine, Sphinganine. **b** The EIC of aligned spot acetyl carnitine metabolites, L-Palmitoylcarnitine, Dodecanoylcarnitine, Palmitoylcarnitine, 2-Methylbutyroylcarnitine.

Figure 4. The LCMSMS spectrum of Sphingosins, including sphingosine, (4E,8E,10E-d18:3)sphingosine, sphingosine 1-phosphate, Phytosphingosine, Sphinganine.

Figure 5 The brain heat map was used to display the screened other representative metabolites in brain. Colors are annotated according to the peak intensities of metabolites in basal ganglia, with red for high concentrations and green for low concentrations.

Table list

Table 1 Different components on locomotion speed, locomotion length and central activity time in mice ($\bar{x}$ ± s， n = 10)

Table 2 Different components on natural sleep duration within 12 hours, sleep latency time and sleep time induced by pentobarbital sodium in mice ($\bar{x}$ ± s, n = 10)

Table 3 The effect of MCI column elution fraction on locomotion speed, locomotion length and central activity time in mice (x ± s, n = 10)

Table 4 The effect of MCI column elution fraction on natural sleep duration within 12 hours, sleep latency time and sleep time induced by pentobarbital sodium in mice (x ± s, n = 10)

Table 5 The effect of monomer compounds on locomotion speed, locomotion length and central activity time in mice (x ± s, n = 10)

Table 6 The effect of monomer compounds on natural sleep duration within 12 hours,

sleep latency time and sleep time induced by pentobarbital sodium in mice ($x \pm s$, n = 10)

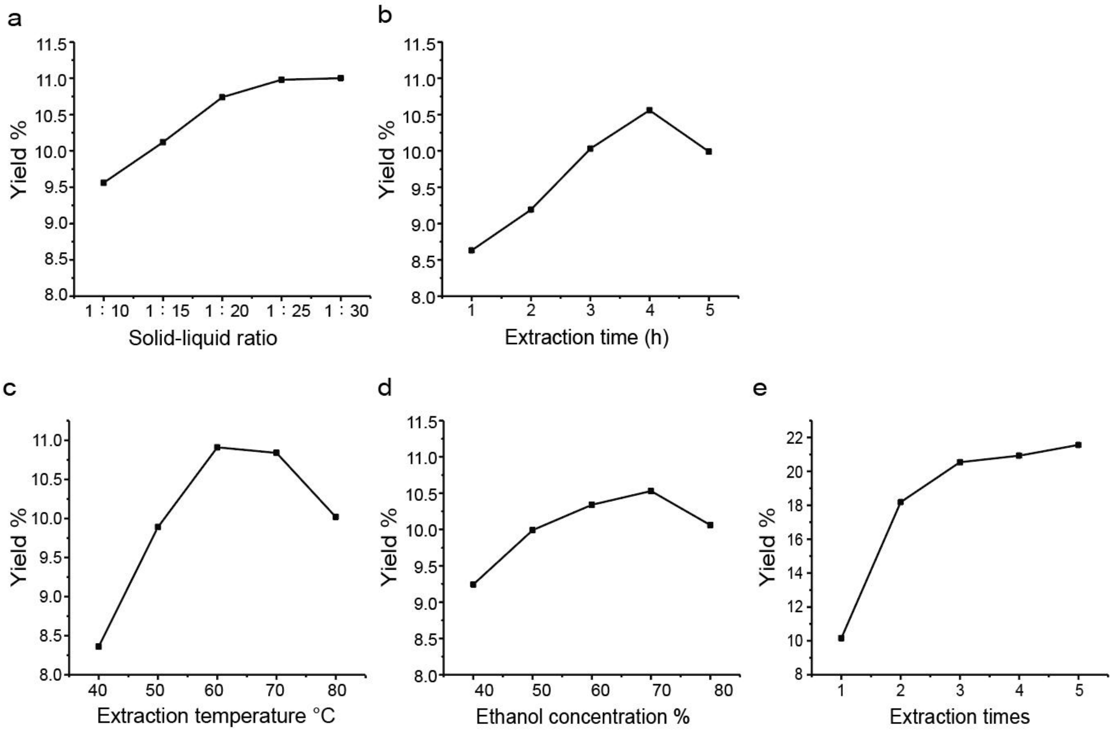

Figure. 1 The optimized extraction parameters of active ingredient of *diaphragma juglandis fructus*. **a** The influence of material-to-liquid ratio on the yield of crude extract. **b** The influence of extraction time on the yield of crude extract. **c** The influence of extraction temperature on the yield of crude extract. **d** The influence of ethanol concentration on the yield of crude extract. **E** The influence of number of extractions on the yield of crude extract

It can be seen from **Fig. 1a** that with the increase of the solid-liquid ratio, the yield of the extract gradually increases, which may be because the contact area between the material and the extraction solvent gradually increases. However, when the ratio of solid to liquid exceeds 1:20, the increase of yield tends to be flat, which may be because the contact area has reached the maximum value. In order to save costs, it is better to choose a material-liquid ratio of 1:10. It can be seen from **Fig. 1b** that within 1~4 h, the yield increases with the extension of the extraction time, and reaches the maximum value at 4 h. When it exceeds 4 h, the yield begins to decrease. It may be because the extraction time is too long that the ethanol is volatilized, which leads to the precipitation of some substances that are slightly soluble in ethanol, and finally affects the yield. With the increase of temperature, the extraction yield of the extract showed a trend of first increase and then decrease, in **Fig. 1c**. When the temperature is lower than 60 °C, the yield gradually increases with the increase of temperature, which is because the solubility of some substances increases with the increase of temperature; when the temperature is higher than 60 °C, the yield decreases obviously, which is Some thermally unstable substances are easily decomposed when the temperature is too high. Comprehensively, the extraction temperature was selected as 60°C. In **Fig. 1d,** between the range of 40%~70% ethanol concentration, the extraction yield of the extract increases with the increase of ethanol concentration, and reaches the maximum at 70% concentration; when

the ethanol concentration exceeds 70%, the yield The possible reason is that the boiling point of the solution decreases due to the high concentration of ethanol, so a large amount of ethanol is volatilized, causing partial dissolution of substances slightly soluble in ethanol, so the yield decreases. It can be seen from **Fig. 1e** that the extraction yield of the extract increases with the extraction times, and the extract yield does not increase much after 2 times. Considering the cost and yield, it is better to choose the extraction times 2 times.

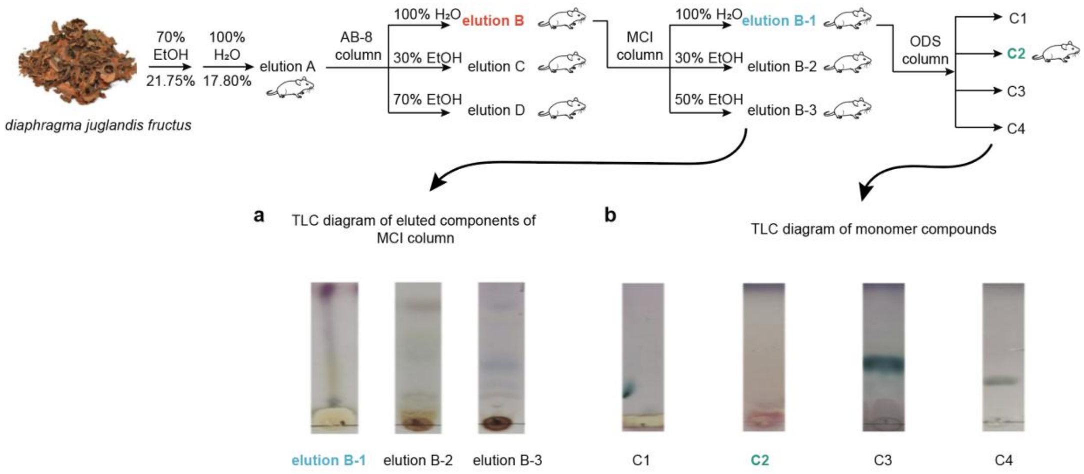

Figure 2 The TLC diagram of eluted components of MCI column and monomer compounds. **a** Three eluted components of MCI column. **b** Four monomer compounds.

a

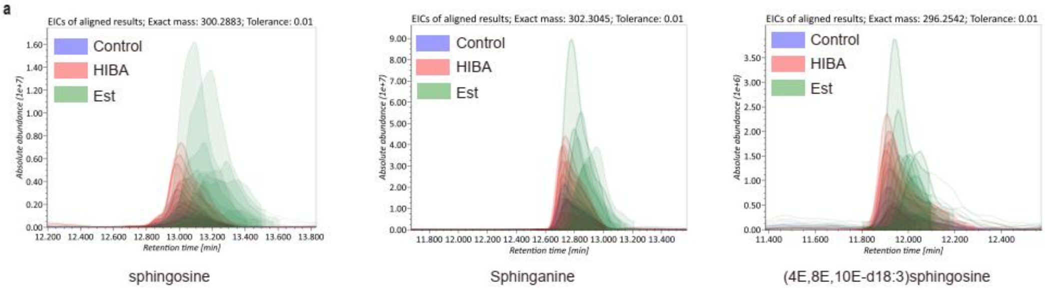

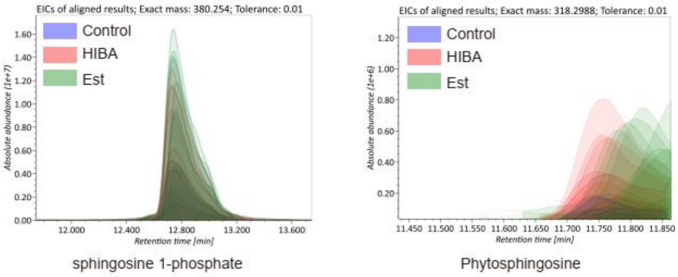

Metabolites	pvalue	FC
sphingosine	0.0035	3.29
Sphinganine	0.0039	2.20
(4E,8E,10E-d18:3)sphingosine	0.003	3.2
sphingosine 1-phosphate	0.004	3.2
Phytosphingosine	0.0086	3.2

b

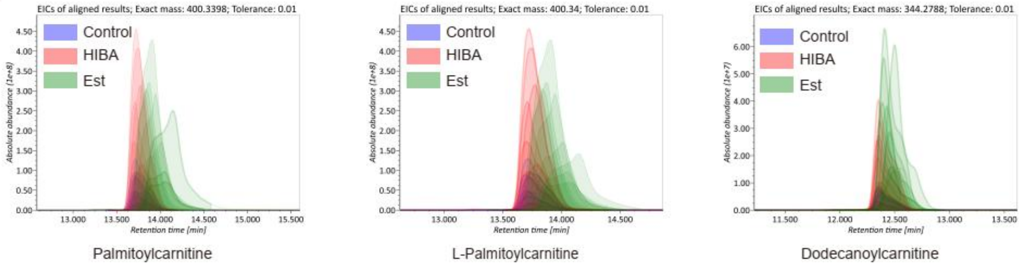

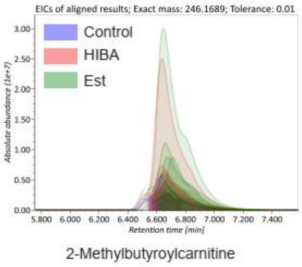

	pvalue	FC
Palmitoylcarnitine	0.04	2.45
L-Palmitoylcarnitine	0.01	4.2
Dodecanoylcarnitine	0.006	3.8
2-Methylbutyroylcarnitine	0.15	2.2

Figure 2. The EIC of aligned spot of BG metabolites in mice brain treated with HIBA (n = 8) and Est (n = 8). **a** The EIC of aligned spot Sphingosine like metabolites, sphingosine, (4E,8E,10E-d18:3)sphingosine, sphingosine 1-phosphate, Phytosphingosine, Sphinganine. **b** The EIC of aligned spot acetyl carnitine metabolites, L-Palmitoylcarnitine, Dodecanoylcarnitine, Palmitoylcarnitine, 2-Methylbutyroylcarnitine.

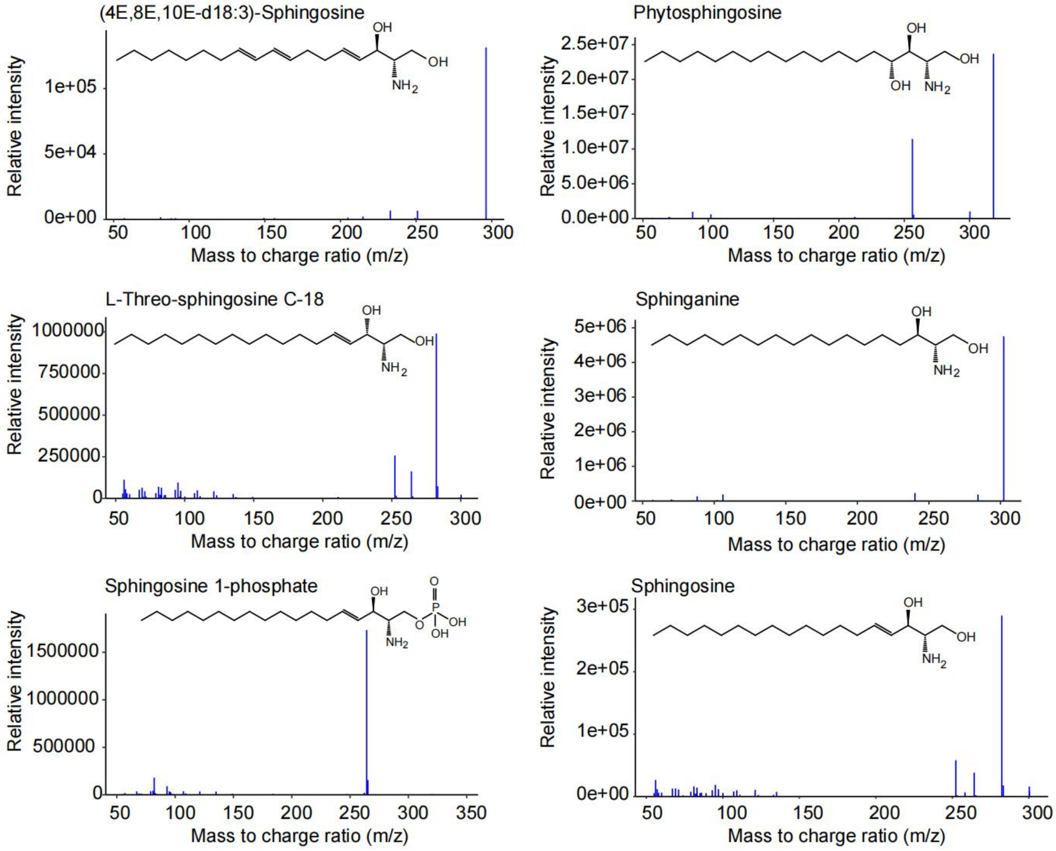

Figure 3. The LCMSMS spectrum of Sphingosins, including sphingosine, (4E,8E,10E-d18:3)sphingosine, sphingosine 1-phosphate, Phytosphingosine, Sphinganine.

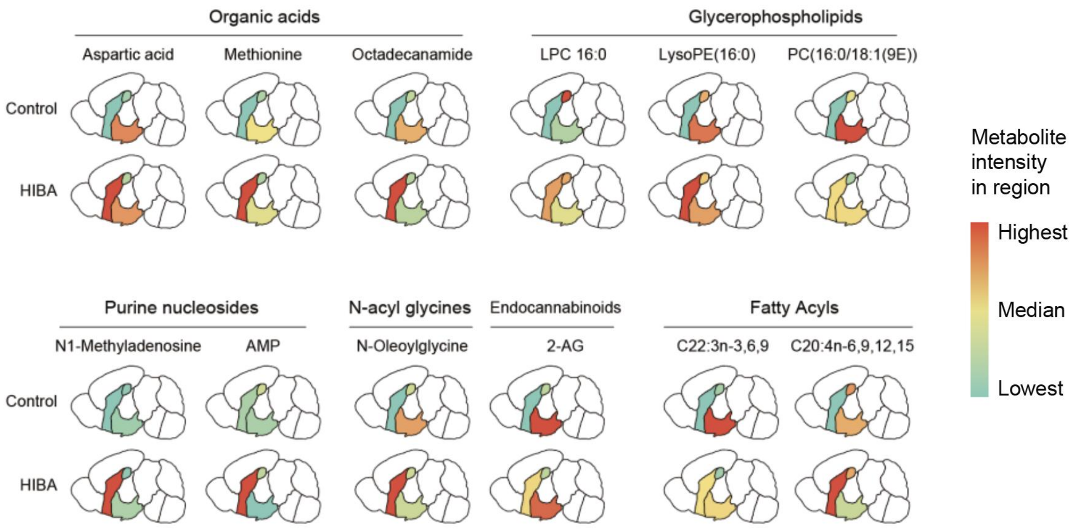

Figure 4. The brain heat map was used to display the screened other representative metabolites in brain BG. Colors are annotated according to the peak intensities of metabolites in basal ganglia, with red for high concentrations and green for low concentrations.

It could be noticed that in basal ganglia, the 5 glycerophosphocholines metabolites: 1-Palmitoylphosphatidylcholine, LPC 16:0, LysoPE(16:0), LysoPE(18:0), PC(16:0/16:0), 2-Oleoyl-1-palmitoyl-sn-glycero-3-phosphocholine, PC (16:0/18:1(9E)), were all upregulated. Furthermore, glycerylphosphorylcholine increases growth hormone and GABA, as brain's natural calming agent. These glycerylphosphorylcholines increased in basal ganglia, indicating that the small molecule HIBA may regulate the expression of Glycerophosphocholine through metabolism, and then promotes the expression of GABA level, thereby promoting sleep.

Table. 1 Different components on locomotion speed, locomotion length and central activity time in mice ($\bar{x} \pm s$，n = 10)

Groups	locomotion length (cm/s)	Locomotion time (s)	central activity time %
Control	5.26±0.66	347.33±49.92	12.55±5.18
A	5.35±0.69	340.27±40.78	13.50±8.25
**B**	**4.08±0.46***	**283.79±19.59***	**18.20±7.82**
C	5.58±0.92	385.88±50.20	15.24±4.41
D	4.30±0.31	302.81±30.54	15.52±7.19
Est	3.94±0.81*	254.53±62.81**	22.93±8.33*

* p<0.05，** p<0.01

Table. 2- Different components on natural sleep duration within 12 hours, sleep latency time and sleep time induced by pentobarbital sodium in mice ($\bar{x} \pm s$, n = 10)

Groups	natural sleep duration within 12 hours (min)	sleep latency time (min)	sleep time (min)
Control	240.21±32.03	4.91±0.93	15.72±5.32
A	280.03±24.73	4.01±0.97	19.67±5.23
**B**	**352.44±23.11****	**3.70±0.42***	**25.39±7.17***
C	271.84±24.30	4.64±1.44	14.49±5.42
D	269.35±23.00	4.43±0.75	20.83±6.37
Est	402.44±27.01**	3.64±0.80*	55.71±10.90**

* p<0.05，** p<0.01。

Table. 3 The effect of MCI column elution fraction on locomotion speed, locomotion length and central activity time in mice ($x \pm s$, n = 10)

Groups	locomotion length (cm/s)	Locomotion time (s)	central activity time %
Control	4.63±0.94	335.84±54.68	10.73±2.11
**B-1**	**3.89±1.04**	**278.18±52.86***	**16.93±7.64***
B-2	4.16±0.95	298.25±66.94	10.60±3.01
B-3	4.20±0.79	293.22±46.83	10.40±2.00
Est	3.39±0.50*	245.21±38.93*	19.36±8.77*

* p<0.05， ** p<0.01。

Table. 4 The effect of MCI column elution fraction on natural sleep duration within 12 hours, sleep latency time and sleep time induced by pentobarbital sodium in mice ($x \pm s$, n = 10)

Groups	natural sleep duration within 12 hours (min)	sleep latency time (min)	sleep time (min)
Control	256.22±36.91	4.86±0.98	16.32±2.94
**B-1**	**349.47±39.10***	**3.79±0.56***	**24.33±6.03***
B-2	286.93±25.57	4.84±1.07	17.51±5.85
B-3	238.62±25.19	4.64±1.00	16.99±7.11
Est	383.73±20.29**	3.53±0.54*	62.23±9.88**

* p<0.05， ** p<0.01

Table. 5 The effect of monomer compounds on locomotion speed, locomotion length and central activity time in mice (*x* ± s, n = 10)

Groups	locomotion length (cm/s)	Locomotion time (s)	central activity time %
Control	6.23±1.27	421.20±63.41	9.80±2.34
C1	5.05±0.96	377.48±29.99	11.35±3.14
**C2**	**4.66±1.14***	**333.76±56.84***	**17.81±5.14****
C3	5.55±1.83	381.19±55.30	12.65±2.47
C4	6.13±2.00	389.74±44.68	12.14±4.40
Est	4.60±0.96*	325.85±51.64*	20.63±9.01**

* p<0.05， ** p<0.01

Table. 6 The effect of monomer compounds on natural sleep duration within 12 hours, sleep latency time and sleep time induced by pentobarbital sodium in mice (*x* ± s, n = 10)

Groups	natural sleep duration within 12 hours (min)	sleep latency time (min)	sleep time (min)
Control	289.89±31.54	4.54±0.75	17.92±4.57
C1	347.48±27.44*	4.33±0.96	33.51±10.75**
**C2**	**374.33±24.48****	**3.60±0.37****	**34.73±10.63****
C3	302.80±25.80	4.70±0.97	21.81±10.63
C4	291.47±29.88	3.61±0.49*	15.79±3.85
Est	404.10±34.03**	2.81±0.57**	66.37±21.05**

Table. 5 Standard curve line, linear ranges, regression equations, correlation coefficients

Neurotransmitters	Rt (min)	Calibration curve	R^2	ng/mL	LOQ (ng/mL)
Ach	1.63	y=87856x-66796.3	0.9997	4-500	0.8
5-HT	2.24	y=12712x+49477	0.9995	4-500	0.8
DA	2.82	y=57778x+593.32	1.0000	4-500	0.8
GABA	4.30	$y=5795.9x+10^7$	0.9900	40-5000	8
Glu	5.93	y=5061.7x+304868	0.9980	40-5000	8
Asp	6.38	y=217.23x+21558	0.9936	200-50000	4
Gly	5.44	y=117.88x+27911	0.9935	40-5000	8
Tau	4.62	y=433.13x+143133	0.9978	200-50000	4

and lower limit of quantitation of eight neurotransmitters
